# The Individualised versus the Public Health Approach to Treating Ebola

**DOI:** 10.1371/journal.pmed.1001858

**Published:** 2015-07-28

**Authors:** Tom H. Boyles

**Affiliations:** Division of Infectious Diseases and HIV Medicine, Department of Medicine, University of Cape Town, Cape Town, South Africa; King’s Sierra Leone Partnership, King’s Centre for Global Health, King’s College London, and King’s Health Partners, London, United Kingdom

## Abstract

Tom Boyles reflects on differing approaches taken for treating patients with Ebola virus disease in low- and high-resource settings.

The mortality rate for patients with Ebola virus disease (EVD) in West Africa is approximately 65% [1]. There are no published figures for high-resource settings, but media sources and individual case reports suggest it is much lower and approaches 0% for those who receive this level of care from the beginning of their illness. In their article “Ebola Viral Disease: Experience and Decision Making for the First Cases outside of Africa,” David Stephens and colleagues give insight into the care that can be provided when available resources are not the limiting factor [2]. They describe the decision to open the Serious Communicable Diseases Unit (SCDU) of Emory University Hospital (EUH) when two United States patients contracted EVD while working in West Africa. Using a large specialist team, they provided high-quality care in a safe working environment and disseminated their knowledge and experience widely. In particular, they were able to respond to the huge increase in requests from health care facilities in the US for help in excluding the diagnosis of EVD. Caring for patients using an individualised approach under ideal circumstances contrasts with, but can also inform, the public health approach to care under resource-limited conditions in West Africa. The models of care employed in each environment show some similarities and also have a number of key differences.

## Models of Care

During a generalised outbreak of EVD, a key task is to screen sick patients presenting to health care facilities. Patients suspected of having EVD must be admitted to an isolation unit, which itself must isolate patients from one another until EVD testing is complete. Screening algorithms must have a very high negative predictive value to prevent EVD-positive patients being moved to general wards or discharged home. These units, known as Ebola holding units (EHUs) in Sierra Leone, were hastily constructed, often from very basic materials when sick patients began presenting to health care facilities. As a result, many provide far from ideal working conditions with increased risks of nosocomial transmission.

Once EVD is confirmed, patients can be cohorted together, which means transfer to an Ebola treatment unit (ETU). Some ETUs are part of the same complex as the EHU, but others were built at sites away from health care facilities. Their construction was swift, but they were purpose built and therefore more appropriate for EVD care than many EHUs.

The circumstances were slightly different in the US. There was an increase in suspected cases of EVD in the whole of the US that coincided with the first patients arriving at EUH, but the chance of any given patient being positive was much lower. Rather than creating the equivalent of EHUs, the model was to disseminate knowledge of screening algorithms for use across the country, with only a minority of patients requiring isolation. The model of care at EUH was similar to a West African ETU, with patients having EVD confirmed before arrival and being transferred under the strictest infection prevention and control (IPC) precautions to a pre-existing purpose-built facility.

## Staff Compliment

A leading contrast between West Africa and EUH is the availability of staff resources. Rather than a large team of specialists caring for one or two patients at a time, a typical EHU such as at the Connaught hospital in Freetown, Sierra Leone, had a total staff compliment of around 50 and cared for 16–18 patients simultaneously, with multiple admissions, discharges, and deaths per day. At the height of the epidemic, we estimated total time available to treat a patient was 20 minutes per day [3]. Early in the epidemic, it was estimated that clinical staff had only 1–2 minutes per patient per day to formulate a treatment plan [4]. A key clinical question was how to spend that time most efficiently and in particular how best to administer fluids to patients.

## Fluid Replacement

The predominant clinical features of the EVD outbreak are fever, headache, diarrhoea, and vomiting [4–7], and patients become severely dehydrated, often developing hypovolaemic shock. A small number of centres have been able to routinely measure renal function and found that whereas both hypo- and hypernatraemia occur, hypokalaemia is almost universal and renal impairment is common [7]. Despite the lack of randomised controlled trials, most expert guidance has focussed on the importance of replacing fluids and electrolytes, and a key question is the optimal route of administration. For cooperative patients with mild disease, oral replacement is appropriate and probably lifesaving. For more severe cases with severe diarrhoea, uncontrollable vomiting, or confusion, the ideal route is intravenous, but this presents several challenges. Early experience at the Connaught hospital was of severe adverse events such as haemorrhage, probably related to severe thrombocytopenia, particularly when confused patients removed their cannulas. This represented an infection control hazard as well as an obvious risk to patients. With such limited human resources, it was not possible to intensively nurse or safely sedate patients for this purpose. With so many challenges, few centres have been able to offer intravenous fluid therapy to the standard of an intensive care facility in a high-resource setting, and this has undoubtedly contributed to the higher mortality rate.

Because of the challenges of replacing fluids and electrolytes in resource-constrained settings, one possibility is to use antidiarrheal agents to limit gastrointestinal fluid and electrolyte losses and prevent the problem at its source [8]. A meta-analysis of randomized trials of loperamide in combination with antibiotic therapy for management of infectious diarrhoea in adults has demonstrated its safety and efficacy [9], and there has been some success in using loperamide during the current outbreak [4].

## Biosafety and Waste Disposal

Maintaining high levels of biosecurity in West Africa has been challenging. Supply of personal protective equipment (PPE) was rarely a problem, but facilities for decontamination, particularly in the hastily constructed EHUs, were often far from ideal. The most vulnerable period is while doffing PPE, and great care had to be taken to ensure staff safety under these conditions. Large volumes of dry and wet waste were created at all sites. While some purpose-built EHUs had state-of-the-art incinerators, many smaller units relied on burning waste in pits, which is not an ideal practice.

There is uncertainty regarding optimal IPC precautions for EVD, with each organisation using slightly different procedures and products. It is important that precautions are both effective and resource efficient, which is one area in particular in which experience from centres such as EUH could be used to inform lower-resourced settings.

## Point-of-Care Rapid Diagnostic Tests

Point-of-care rapid diagnostic tests (RDTs) have the potential to revolutionise the screening process for EVD. While sensitive screening algorithms can reduce the volume of patients admitted to EHUs, definitive diagnosis requires a laboratory-based real-time polymerase chain reaction (RT-PCR), which is costly and can have a turnaround time of up to 7 days. Isolating all patients suspected of having EVD is resource intensive; it exposes EVD-negative patients to risk of nosocomial transmission and delays diagnosis and treatment of their underlying condition. A clinically useful RDT must be sensitive enough that patients with negative results can confidently be admitted to general wards or discharged home without risk of onward transmission. The World Health Organization recently endorsed a RDT with sensitivity of 92% that does not meet these requirements and is therefore unlikely to be useful clinically [10]. Fortunately, a second test has now been validated on clinical cases and found to have sensitivity of 100% [11]. This test has the potential to dramatically reduce the patient load at EHUs and free up resources for clinical care.

## Strategies for Treating EVD

Research into treatment and prevention strategies has focused on vaccines, antivirals, and convalescent blood products. While an efficacious vaccine or a cheap and effective antiviral may be beneficial in future outbreaks, it is unlikely that convalescent blood products will ever be practical in low-resource settings even if they prove efficacious. In testing such strategies, resources have been diverted from trials to optimise the standard of care using easily available treatments such as antidiarrhoeal agents, antibiotics, and electrolyte replacement. With the end of the outbreak in sight, the opportunity to test these cheap and practical strategies has passed for now.

## Conclusion

Caring for patients with EVD using an individualised approach in high-resource settings reduces mortality towards zero. Mortality is much higher in low-resource settings where a public health approach is needed. In such settings, it is vital to use resources in the most efficient way possible to tip the balance in favour of survival for as many patients as possible.

## Abbreviations

EHU: Ebola holding unit
ETU: Ebola treatment unit
EUH: Emory University Hospital
EVD: Ebola virus disease
IPC: infection prevention and control
PPE: personal protective equipment
RDT: rapid diagnostic test
RT-PCR: real-time polymerase chain reaction
SCDU: Serious Communicable Diseases Unit
